# Cardiology career satisfaction: a little academic activity goes a long way

**DOI:** 10.3389/fcvm.2024.1385509

**Published:** 2024-03-20

**Authors:** Christopher R. deFilippi

**Affiliations:** Inova Schar Heart and Vascular Institute, Falls Church, VA, United States

**Keywords:** clinical cardiologist, burnout—professional, research learning, academic performance, career satisfaction

## Abstract

The professional landscape for clinical cardiologists and most physicians has changed dramatically in the last decade in the United States. By the end of 2020, 87% of cardiologists were integrated with a health system (employed or part of a professional services agreement). Physicians transitioning to a large employer are often dissatisfied with the lack of autonomy and the pressure from “one-size-fits-all” productivity targets. The results from physician surveys indicate that physicians practicing clinically in an academic environment have greater job satisfaction. Potentially even a modest amount of time comprising 10-20% of total effort spent on academic pursuits that are most meaningful to the individual physician can result in nearly a two-thirds lower risk of burnout compared with physicians who don't receive this time. The opportunity to participate in this special topic compendium by cardiovascular specialists at one regional integrated health system in the United States is an example of an opportunity to successfully incorporate meaningful professional academic opportunities into a clinical care environment.

The professional landscape for clinical cardiologists and most physicians has changed dramatically in the last decade in the United States. By the end of 2020, 87% of cardiologists were integrated with a health system (employed or part of a professional services agreement) (1). The Inova health system, located in northern Virginia outside of Washington, DC, is consistent with this trend. Physicians transitioning to a large employer can feel dissatisfaction from a lack of autonomy, pressure from “one-size-fits-all” productivity targets, and outcomes judged by patient satisfaction scores. Productivity demands at their worst can lead to the phenomenon of “burnout,” with a paradox that higher demands with greater clinical productivity can lead to a lower sense of personal accomplishment. There can be serious consequences of “burnout.” A recent meta-analysis showed that a sense of lower personal accomplishment results in 47% more patient safety incidents adversely impacting all within a health system (2). How then might this be addressed in a constructive manner to promote professional wellbeing, expertise, and autonomy with recognition that health systems often work with razor-thin financial margins? Thinking as a clinician, one considers both a peer-reviewed evidence base and anecdotal experience to address this issue.

Physicians, in general, including those who predominantly do procedures, appear to have greater career satisfaction in an academic environment than in non-affiliated practices despite similar work hours (3, 4). From the 2019 Association of American Medical College's (AAMC) survey, which included 40% academically affiliated physicians, academically affiliated physicians reported 13% less feelings of burnout, and more satisfaction with time use and their career compared with unaffiliated physicians (4). In another survey of 3,170 graduates from one US medical school, those more involved in teaching, research, and lifelong learning activities had higher career satisfaction (5). Those with the highest career satisfaction were also the most likely to be engaged professionally through the publication of papers, presentations at meetings, serving on professional committees, and playing a role with medical journals (5). It turns out that only a modest amount of academic time (reflected by teaching, research, and administrative leadership roles) might make a big difference. Another survey study of physicians at a large academic medical center where over two-thirds reported patient care as the most meaningful aspect of their work found that physicians allowed just 10%–20% of their time to do what was most meaningful to them professionally were 64% less likely to experience burnout than those not provided this time (6). There is a caveat to most of the aforementioned analysis in that they are cross-sectional associations and implying causality (i.e., academic activity improves career satisfaction) is not possible. In fact, the survey of 3,170 graduates from one medical school additionally found that those with the highest career satisfaction also ranked highly in their medical class suggesting that enthusiasm and ability early on guided lifelong career choices and professional engagement (5).

Our anecdotal experience with a recently created Heart and Vascular service line, recruitment of an external academically oriented leader, and consolidation of multiple private practice groups into an employment model took place in the mid-2010s. It became immediately apparent that a cardiology fellowship training program would be critical to defining the culture of the institute. To allay the concerns of the director of a graduate medical education program, over 40 cardiologists (about half of the total privileged staff) showed up to an evening meeting to attest to their commitment to train fellows and in 2018 a general cardiology fellowship was started, now with many attendings competing for time on teaching services. Most of the fellows from this recently started program now go on to prestigious university subspecialty training programs. Many of the attendings who led articles as part of this special issue already participate in professional society committees and coauthor with many others but infrequently take a lead role. Others are known regionally for their expertise on a topic and have developed specialized programs within the health system. In both cases there may be limited venues, particularly including our trainees, to publish on our expertise and practice with our patients. As topic coordinators for this special issue, the immediate enthusiasm of our colleagues to contribute couldn't have been better. We solicited 22 topics, and we got 21 positive responses. More importantly, we then received 21 well-written articles to send forward to the journal for peer review. While coming together as a large group of cardiovascular care providers to create and share our expertise in this special issue certainly cannot solve all the challenges associated with contemporary clinical practice, it added to our broader sense of purpose and brought professional satisfaction to many who participated. We recommend the process highly to others.

## Data Availability

The original contributions presented in the study are included in the article/Supplementary Material, further inquiries can be directed to the corresponding author.
